# Persistent hypoglossal artery detected incidentally in a hypertensive patient with intracerebral hemorrhage: a case report and review of the literature

**DOI:** 10.4076/1757-1626-2-8571

**Published:** 2009-07-24

**Authors:** Serhat Avcu, Irene van der Schaaf, Hatice Nursun Ozcan, Ilker Sengul, Hendrik Fransen

**Affiliations:** 1 Department of Radiology, AZ Sint-Lucas Hospital9000 GentBelgium; 2 Department of Head and Neck Surgery, Gent University9000 GentBelgium

## Abstract

A 73-year-old woman with a history of chronic hypertension admitted to our clinic with complaint of acute paresis in her left arm. Computed tomography and magnetic resonance imaging were performed to the patient which displayed intracerebral hemorrhage in the right parieto-occipital lobe. Further review of the computed tomography and magnetic resonance imaging scan showed an enlarged left hypoglossal canal with a large vessel passing through it. The patient was thereafter examined with cerebral digital subtraction angiography to determine the cause of hemorrhage, but no vascular etiology was demonstrated, except from a persistent hypoglossal artery. We present a rare case of persistent hypoglossal artery, which we detected incidentally during angiographic evaluation, with a review of the literature.

## Introduction

Early in the embryogenesis, multiple anastomoses exist between the carotid and basilar arterial system. Normally, during embryonal development these anastomoses disappear. These connections usually obliterate starting at the fourth week of fetal development, as the posterior communicating arteries develop. In case of failure of involution, patients have a persistent carotid-basilar anastomosis [1-3].

The persistent hypoglossal artery (PHA) is one of four primitive connections between the anterior and posterior circulations that normally regress in utero. Of the four, the persistent trigeminal artery (PTA) makes up the vast majority, with the PHA a distant second. These vessels have some clinical importance when they are associated with aneurysms or arteriovenous malformations, or when carotid endarterectomy is considered, but are generally incidental findings [1]. We present a case of PHA, which we detected incidentally during angiographic evaluation, with the review of the literature.

## Case presentation

**Figure 1.**
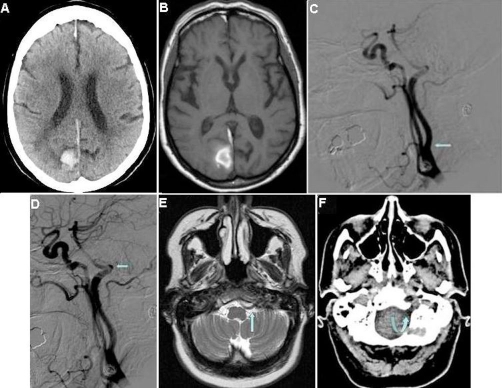
Bleeding in the right parieto-occipital lobe visualized on the non-contrast enhanced CT scan **(A)** and T1 weighted MRI **(B)**. The lateral digital subtraction angiogram shows the hypoglossal artery originating from the carotid artery **(C, arrow)** and its conjunction with the basilar artery **(D, arrow)**. The T2 weighted MR image **(E, arrow)** demonstrates the hypoglossal artery passing through the hypoglossal canal, which is enlarged as shown on the non-contrast enhanced-CT **(F, curved-arrow)**.

A 73-year-old Belgian woman, with a medical history of hypertension, was referred to the hospital with acute paresis of her left arm. A CT scan was performed showing a bleeding in the right parieto-occipital lobe (Figure 1A). Additionally, an MRI scan was made, which confirmed the bleeding but did not show an underlying abnormality that could have caused the bleeding (Figure 1B). The patient underwent digital subtraction angiography (DSA) and again no aneurysm or aberrant intracerebral vessels were detected. However, the DSA revealed a persistent anastomosis between the left internal carotid artery (ICA) and basilar artery and absence of the left vertebral artery and posterior communicating artery. The anastomosis arose from the dorsal side of the ICA in front of cervical body 2 and 3. The artery ended in the basilar artery (Figure 1C, D). On the right side, a hypoplastic vertebral artery was present. Further review of the CT and MRI scan showed an enlarged left hypoglossal canal with a large vessel passing through it (Figure 1E, F), supporting this persistent anastomosis to be a PHA.

## Discussion

The described persistent carotid vertebrobasilar anastomosis (PCVBA) includes type-I and II proatlantal arteries, trigeminal artery, hypoglossal artery, and otic artery. PCVBA form early, during fetal embryogenesis at approximately 24 days. Ordinarily, the otic artery disappears at the 4th week of embryogenesis, followed soon after by the disappearance of hypoglossal and trigeminal arteries. The proatlantal intersegmental maintains the posterior circulation until the vertebral arteries are fully developed between 7 and 8 weeks. When these anastomoses fail to become obliterated, they become congenital persistent anastomoses. The incidence of the PCVBAs has been found to be inversely related to their order of disappearance. In terms of frequency, the trigeminal artery has a reported incidence of 0.1-0.2%, the hypoglossal artery is the second carotid-basilar anastomosis (0.02 to 0.09%) and the primitive otic artery has been convincingly documented only once before, with angiography [2,3].

The distinction of persistent hypoglossal artery and proatlantal artery type-I is critical because the type-I proatlantal artery is far less associated with complications than the hypoglossal artery. No aneurysm has been described on its course and the type-I proatlantal artery does not lead to any symptomatic neurological compression. Besides, the hypoglossal artery is the most common PCVBA associated with hypoplasia of the two vertebral arteries and the circle of Willis. The difference of potential complications between these two arteries may be explained by the fact that the proatlantal artery is only a segmental, somatic artery whereas the hypoglossal artery is an embryonic artery mainly for intra-cranial purpose [6].

The PCVBA in adult patients can be pathological and may result in the development of an aneurysm or a compressive syndrome. The incidence of associated intracranial aneurysms is higher in this population. These anastomoses are frequently associated with proximal or distal arterial pathology [6]. But on the other hand, in their study, Cloft et al [14] claim that the prevalence of cerebral aneurysms in PPTA is no different from the prevalence in the general population; these associations are based solely on case reports, and the coexistence of vascular pathologies with PPTA is quite possibly coincidental. They also point that the PPTA probably is a potential site of aneurysm formation because it is a bifurcation, but it probably has no greater predisposition for aneurysm formation than other bifurcations. They say that PPTA is generally identified by angiography, and if the angiography sessions are often prompted by a symptomatic aneurysm, then there is a bias toward a high prevalence of aneurysms in patients with PPTA who undergo angiography.

The trigeminal, acoustic and type-II proatlantal arteries are easily identified by their origins, whereas the level and location of the ostia of the hypoglossal and type-I proatlantal arteries cannot be considered as a reliable criteria of differentiation. The main criterion to differentiate the latter two anastomoses is the point of entrance in the skull base. On the lateral projection of the digital angiography, the posterior curve of the hypoglossal artery is less important than the strong posterior curve of type-I proatlantal artery, due to its entry into the hypoglossal canal anterior to the foramen magnum [6,7].

The primitive otic artery arises from the carotid artery within the carotid canal, it emerges from the internal acoustic meatus, and it joins the basilar artery at a caudal point [2]. The PTA runs along the trigeminal nerve and anastomoses with the ICA at the C4-5 segments and the basilar artery between the anterosuperior cerebellar arteries and the anterior inferior cerebellar arteries [13].

We describe a case of a left-sided PHA. The hypoglossal artery is bilateral in only 1.4% of cases and slightly more frequent in women and on the left side [6]. This finding corresponds with our case. The PHA arises from the posterior side of the cervical ICA, usually in front of the C1-C2 space but never below the C3-C4 space [6]. In our case, it was in front of the C2-C3 space. It has a short ascending course with a slight medial and posterior curve before penetrating the hypoglossal canal at the skull base, and then ends into the basilar artery.

If the hypoglossal artery persists, the ipsilateral vertebral and posterior communicating arteries are hypoplastic, and the contralateral vertebral and posterior communicating arteries are only present in one-third of the cases [6,8,9]. Thus, the posterior portion of the circle of Willis is inconstant, which makes the hypoglossal artery the exclusive or mainly exclusive feeder of the posterior circulation. This means that temporarily clamping the hypoglossal artery, during carotid endarterectomy, possesses important ischemic risks [8,9]. In those cases, some authors prefer angioplasty, which allows shortening the clamping time [10].

Some clinical symptoms associated with a PHA include a hypoglossal nerve (XII) palsy, as well as a neuralgia of the glossopharyngeal nerve (IX) due to irritation by the large PHA [9,11,12]. However, the PHA is mostly found incidentally at angiographic evaluation, as was the case in our patient in whom, there was no remarkable clinical symptom that could be associated with this finding. Although a few cases about the coexistence of PTA and intracranial hemorrhage have been reported in the literature, as far as we are concerned, such coexistence is not known for PHA. Thus, we suppose that the intracerebral bleeding in our patient was most likely associated with chronic hypertension. On the other hand, some cases with subarachnoidal hemorrhage caused by the rupture of PHA aneurysm have been reported.

In conclusion, since the hypoglossal artery is more frequently associated with complications than the type-1 proatlantal artery, and temporarily clamping the hypoglossal artery during carotid endarterectomy, possesses important ischemic risks, it is important to make a proper differential diagnosis.

## Abbreviations

CT: computed tomography
DSA: digital subtraction angiography
ICA: internal carotid artery
MRI: magnetic resonance imaging
PCVBA: persistent carotid vertebrobasilar anastomosis
PHA: persistent hypoglossal artery
PPTA: persistent primitive trigeminal artery
PTA: persistent trigeminal artery
